# Genotype-Specific Growth and Proteomic Responses of Maize Toward Salt Stress

**DOI:** 10.3389/fpls.2018.00661

**Published:** 2018-05-30

**Authors:** Ana L. C. Soares, Christoph-Martin Geilfus, Sebastien C. Carpentier

**Affiliations:** ^1^Laboratory of Tropical Crop Improvement, Division of Crop Biotechnics, KU Leuven, Leuven, Belgium; ^2^Controlled Environment Horticulture, Faculty of Life Sciences, Albrecht Daniel Thaer-Institute of Agricultural and Horticultural Sciences, Humboldt University of Berlin, Berlin, Germany; ^3^Genetic Resources, Bioversity International, Leuven, Belgium; ^4^SYBIOMA, KU Leuven, Leuven, Belgium

**Keywords:** salt sensitivity, maize, physiological phenotyping, proteomics, abiotic stress, antioxidation, class III peroxidases

## Abstract

Salt stress in plants triggers complex physiological responses that are genotype specific. Many of these responses are either not yet described or not fully understood or both. In this work, we phenotyped three maize genotypes of the CIMMYT gene bank alongside the reference B73 genotype (NCRPIS – United States) under both control and salt-stressed conditions. We have ranked their growth potential and we observed significant differences in Na^+^ and Cl^-^ ion accumulation. Genotype CML421 showed the slowest growth, while CML451 had the lowest accumulation of ions in its leaves. The phenotyping defined the right timing for the proteomics analysis, allowing us to compare the contrasting genotypes. In general 1,747 proteins were identified, of which 209 were significantly more abundant in response to salt stress. The five most significantly enriched annotations that positively correlated with stress were oxidation reduction, catabolic process, response to chemical stimulus, translational elongation and response to water. We observed a higher abundance of proteins involved in reactions to oxidative stress, dehydration, respiration, and translation. The five most significantly enriched annotations negatively correlated with stress were nucleosome organization, chromatin assembly, protein-DNA complex assembly, DNA packaging and nucleosome assembly. The genotypic analysis revealed 52 proteins that were correlated to the slow-growing genotype CML421. Their annotations point toward cellular dehydration and oxidative stress. Three root proteins correlated to the CML451 genotype were annotated to protein synthesis and ion compartmentalization. In conclusion, our results highlight the importance of the anti-oxidative system for acclimatization to salt stress and identify potential genotypic marker proteins involved in salt-stress responses.

## Introduction

Salinity is a key cause of arable land loss (Gong et al., 2014). It is estimated that 19.5% of irrigated lands, which provide 40% of the food production worldwide (FAO, 2015), are salt-affected (FAO, 2016). Considering the exponential growth of the population (Melorose et al., 2015), plus the aggravation of the environmental situation by climate change (IPCC, 2007), the challenge is set: increase food production per unit area of cultivated land from more sustainable production systems.

Globally, 52% of human nutrition relies on cereals including maize (FAO, 2015). In general, maize (*Zea mays*) is considered moderately sensitive to salt (Zörb et al., 2004), a category which comprises plants that maintain growth in saline soils with an ECe between 3 and 6 dS m^-1^ (Hasanuzzaman et al., 2013). However, few salt-tolerant cultivars have been commercialized (Flowers and Flowers, 2005).

Increased access to maize genetic diversity (Barros et al., 2010) can provide several alleles linked to desirable tolerance traits. Yet, these alleles are highly dispersed and joint efforts must be made to make the information available for application in biotechnology and breeding programs.

Dynamic phenotyping should precede and guide genomic studies (Zhu, 2002; Zivy et al., 2015) and will be critical in cases where the effects and responses have a dynamic nature, such as salt stress (Cattivelli et al., 2008). Phenotyping at different levels can help validate results from gene expression studies (Zhu, 2002).

Saline effects in plants occur with the combination of two stress phases: first, the osmotic, and second the ionic specific phase (Munns, 1993). For this, tolerance mechanisms are also virtually segmented according to those two phases. Lowering tissue water potential using proline and other compatible solutes contributes is a way to establish osmotic-stress tolerance (Meloni et al., 2001). Stress tolerance mechanisms during the ionic phase can include: ion exclusion, tissue tolerance (Munns and Tester, 2008) and directing sodium ions from the shoot back to the root via the phloem (Tester and Davenport, 2003). The use of each mechanism, aggregated or not, and its effectiveness, are the distinguishing factors between salt-sensitive and salt-tolerant plants (Munns and Tester, 2008).

Salt-tolerant species generally compartmentalize the accumulated ions away from the sites of primary photosynthesis and expanding tissues. The ions are stored in vacuoles and compatible solutes counterbalance the osmotic disequilibrium (Munns, 2002; Munns and Gilliham, 2015). However, is this the most efficient stress-management strategy for crop plants, when aiming at high yield? Will the benefits of the compartmentalization overcome the energy costs for these plants? The necessary investigations of salt-stress signaling and pathways of response will help answer these questions.

Researchers have been active in addressing these questions. A root proteomics study of maize seedlings exposed to 150 mM NaCl stress showed that the responses were quanti- and qualitatively different between a sensitive and a tolerant inbred line (Cheng et al., 2014). The authors suggested that an enhanced antioxidative defense system, allied to other processes, are the fighting arms of tolerant lines. Zörb et al. (2010) identified proteins related to cell wall growth regulation in an early salt stress response of a maize salt-tolerant hybrid. We showed recently that chloride-salinity stress induced in maize leaves stiffened the cell wall, via an apoplast alkalization process (Geilfus et al., 2017). Stiffening of the cell wall would benefit water stressed plants by diminishing water loss and wilting (Kutschera and Schopfer, 1986). Geng et al. (2013) found that the presence of abscisic acid (ABA) and the root endodermis act as a guard to prevent the plant from growing into salinized environments. With proteins having so many significant functions which help confer salt-tolerance to plants, proteomics and the role of proteins in overcoming salinity effects are an important topic of study (Kosová et al., 2013).

Despite many efforts, there are gaps in our understanding of salt stress response pathways, especially for responses by different specific genotypes. We hypothesize that (i) each genotype is capable to respond to the stress and (ii) that phenotyping at whole plant level guides the cellular analyses in identifying specific physiological events. In combining these hypotheses, we therefore used a system-levels approach to characterize salt stress in four different genotypes. We are using the dynamics of the plant responses to get an insight into the reactions at the cellular level. We bring up the challenging definition of tolerance in agriculture and we open a discussion about energy costs and possible introduction of salt-tolerance mechanisms.

## Materials and Methods

The current experiment was held in the months of February and March 2016. In total, four maize genotypes were analyzed: CML421, CML448, and CML451 obtained from the International Maize and Wheat Improvement Center (CIMMYT); and the sequenced reference B73, acquired from the North Central Regional Plant Introduction Station (NCRPIS, United States).

### Growth Conditions and Salinity Treatment

Plants were grown for 4 weeks after germination in a greenhouse, in four-liter pots, filled with sand and DCM potting soil Type 3 mix (2:1), in a 12 h photoperiod and temperature of 25°C/15°C, day and night, respectively. Two experimental conditions were set in a completely randomized design, in which the treatment consisted of sodium chloride (NaCl) addition directly to the soil mix (EC = 9.5 dS cm^-1^), and the control, without the salt addition (EC = 1 dS cm^-1^). All the seeds were sown directly in the soil mix, so the treated ones germinated in the salinized soil. Electrical conductivity (EC) was determined through soil water suspensions (SWSs), with 1:5 soil to water ratio (EC_1:5_). Results of SWS method can be converted to saturated extract paste method (SP) form, according with the type of soil used. In this study, the appropriate regression equation used for conversion from EC_1:5_ to ECe was: SP = 7.98 SWS, with the EC in dS m^-1^ at 25°C (Sonmez et al., 2008).

The experimental design avoided water leaking and guaranteed that the salt was not washed out from the pots, by calculating the exact volume of water solution for each plant to be irrigated. Extreme salt concentration, caused by evaporation of soil water, was also prevented by covering the soil with foil. Each genotype counted with six replicates. The nutrient solution that was used for irrigation had the following concentration: 2 mM KH_2_PO_4_, 1 mM K_2_SO_4_, 1.33 mM Ca(NO_3_)_2_, 0.67 mM NH_4_NO_3_, 2 mM CaCl_2_, 0.5 mM MgSO_4_, 0.1 g L^-1^ Fe-sequestrene, 5 μM H_3_BO_3_, 2 μM MnSO_4_, 0.5 μM ZnSO_4_, 0.3 μM CuSO_4_, 0.01 μM (NH_4_)_6_Mo_7_O_24_.

### Physiological Phenotyping

As soon as the plants started to germinate, the projected leaf area was measured for all plants in each of the six replicates per genotype. Pictures were taken weekly and individually from the top of the plants (Supplementary Figure S1). The green area was obtained by processing the pictures off-line using an in-house script (R Development Core Team, 2003). A quadrant plot, constructed according to Kissel et al. (2015), ranks the genotypes by leaf area of each individual genotype, normalized to the median leaf area of all genotypes. Thus, the division into four quadrants indicates the best and worst potential for the genotypes’ growth, according to the environmental conditions.

The investigation of root growth was estimated by determining the fresh weight of six samples per treatment and per genotype at the fourth week. For these measurements, whole plants were sacrificed and weighed, including the roots, where soil remnants were washed out with water. Growth retardation was calculated for each genotype in terms of changes in leaf area and root mass using the formula: 1 - (average control/average stress) × 100.

The analysis of ions was performed on 20 mg of dried leave material suspended and boiled for 5 min in 1.6 ml of deionized water. After cooling, samples were centrifuged and the supernatant was collected. Subsequently, proteins were precipitated by washes in chloroform. Thereafter, samples were cleaned by passage through strata C-18 columns (Thermo Fisher Scientific, Darmstadt, Germany). Na^+^, K^+^ and Cl^-^ concentrations were analyzed using ion chromatography (Dionex ICS-5000+ Capillary HPIC System, Thermo Fisher Scientific).

### Protein Extraction, Digestion, and Cleaning of Peptides

Roots from the plants sacrificed at the fourth week were cleaned with distilled water and immediately plunged in liquid nitrogen (LN). The protein extraction was realized according to the phenol method described by Carpentier et al. (2005), with 300 mg of LN triturated material from each of the six replicates per genotype and for each treatment. The homogenate was solubilized in 750 μL extraction buffer ice cold (100 mM Tris-HCL pH 8.3; 5 mM EDTA; 100 mM KCL; 1% p/v DTT; 30% sucrose; 1 complete Mini EDTA-free protease inhibitor cocktail tablet -Roche Applied Science- per 10 ml buffer. After briefly vortexing, 750 μL ice-cold Tris buffered phenol (pH 8.0) solution was added and vortexed for 10 min, at 4°C before it was centrifuged at 4°C and 12,000 rpm, for 10 min. The phenolic phase was collected and once more 750 μL buffered phenol was added, the mixture was vortexed and centrifuged for 5 min. After collecting the phenolic phase, 100 mM ammonium acetate in methanol was added and it was kept in -20°C overnight to precipitate. The next day, the samples were directly centrifuged for 60 min (4°C, 13,000 rpm). The supernatant was discarded, and the pellet was rinsed twice with cold acetone and 0.2% DTT solution, being kept in this solution for 60 min at -20°C each time, with centrifugation (4°C, 13,000 rpm) and discard of supernatant in between. The pellet was gently dried under a hood at room temperature, and was resuspended with 150 μL lysis buffer (8 M urea; 5 mM DTT; 30 mM Tris). Protein concentration was quantified with the 2-D Quant Kit assay (GE Healthcare).

Digestion of sample aliquots containing 20 μg of proteins was realized with successive addition of the following compounds, to the following final concentrations: DTT to 20 mM and incubated for 15 min; iodoacetamide to 50 mM, incubation in the dark for 30 min; three times dilution with 150 mM ammonium bicarbonate; addition of trypsin in a ratio of 0.2 μg trypsin per 20 μg protein and overnight incubation in 37°C; and acidification with trifluoroacetic acid to 0.1% final concentration. Desalting of samples was performed using Pierce^TM^ C18 Spin Columns (Thermo Fisher Scientific), according to manufacturer instructions.

### Peptides Separation, Identification, and Quantification

Digested samples (1 μg/5 μL) were separated via UPLC-MS/MS system, in which the Ultimate 3000 UPLC system (Dionex, Thermo Scientific) and the Q Exactive Orbitrap mass spectrometer (Thermo Scientific, United States) were used according to Vanhove et al. (2015). Data were obtained with Xcalibur 3.0.63 software (Thermo Scientific). Protein identification was realized with the conversion of the raw data by Proteome Discover version 1.4 (Thermo Scientific) into mgf files and processing with MASCOT version 2.2.06 (Matrix Science) against the Uniprot *Zea mays* database (99 371 proteins). Calculation of false discovery rate (FDR) was realized with Scaffold (Version: Scaffold 3.6.3; Proteome Software Inc., Portland, OR, United States). Quantification of peptides was determined using Progenesis LC-MS version 4.1 (Nonlinear Dynamics), with automatic alignment from a selected reference run. Abundance of proteins was based on sum of peptides quantification. Singular enrichment analysis (SEA) was performed for selected proteins using the AgriGO SEA tool^1^, with *Zea mays* background and significance level of 0.01. Promoter analysis was conducted using plantCARE (Lescot et al., 2002), up to 1,500 bp and using genotype B73 as the sequenced reference.

### Statistics

For both physiological and cellular phenotyping, statistics were performed through the software STATISTICA (version 13, Tulsa, OK, United States). After an ANOVA analysis, the means were compared with test Fisher’s LSD (*p* < 0.05). Box plots are composed by the median, the box is defined by the first and third quartiles. Whiskers are positioned 1.5 times the interquartile range from the median. Individual points distancing more from the median than the extremes are the outliers. Quadrant plot was constructed in Microsoft Excel (Office 2016), according to Kissel et al. (2015). *N* = 6 for top area, fresh weight, EC and proteomics analysis. For ion content, *N* = 4.

## Results

### Physiological Phenotyping Toward NaCl Effects

**FIGURE 1:**
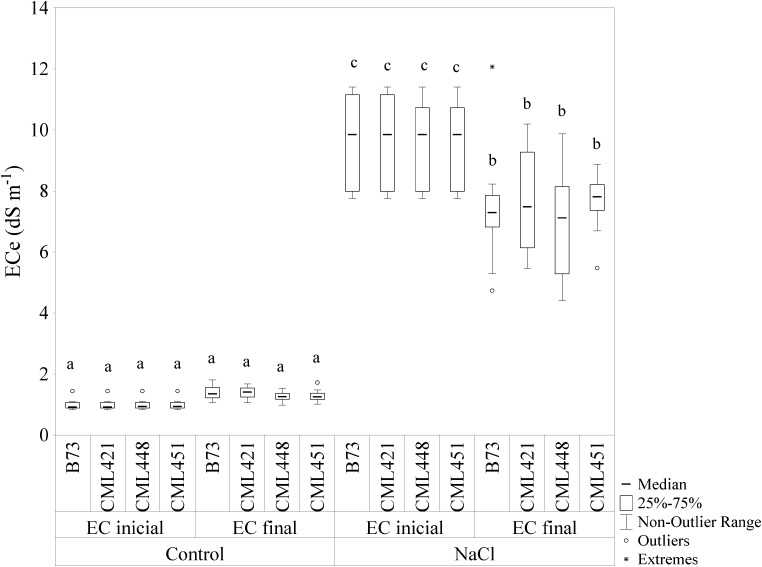
Electric conductivity (EC) of the soil mix with addition of NaCl or not (control). The EC was calculated by soil water suspensions (SWSs), with 1:5 soil to water ratio (EC_1:5_) and the values were converted using a regression equation, with the final EC in dS m^-1^ at 25°C. Soil samples were measured at the beginning (EC_inicial_) and at the end (EC_final_) of the experiment. Means followed by the same letter did not differ according to the LSD test (*p* ≤ 0.05). *N* = 6.

The initial and final EC (EC_i_ and EC_f_) for the control soil were 1 dS cm^-1^ and 1.3 dS cm^-1^, respectively (**Figure 1**). For saline soil, the values were EC_i_ = 9.8 dS cm^-1^ and EC_f_ = 7.4 dS cm^-1^. The EC of saline soil showed a significant difference in the median, with a slight decrease from the initial to the final EC.

Physiological phenotyping of the four maize genotypes was performed not only to secure a link between agronomical effects of the salt stress and cellular phenotyping, but also to determine a specific time for sample collection. Therefore, seedlings were monitored weekly, by means of top images (Supplementary Figure S2). At week 4, destructive samples were taken of the leaves, used for determination of the ion content, and of the roots to perform the proteome analysis. The first significant difference in projected leaf area between salt treated and non-treated plants occurred from the third to the fourth week after sowing (Supplementary Figure S2).

**FIGURE 2:**
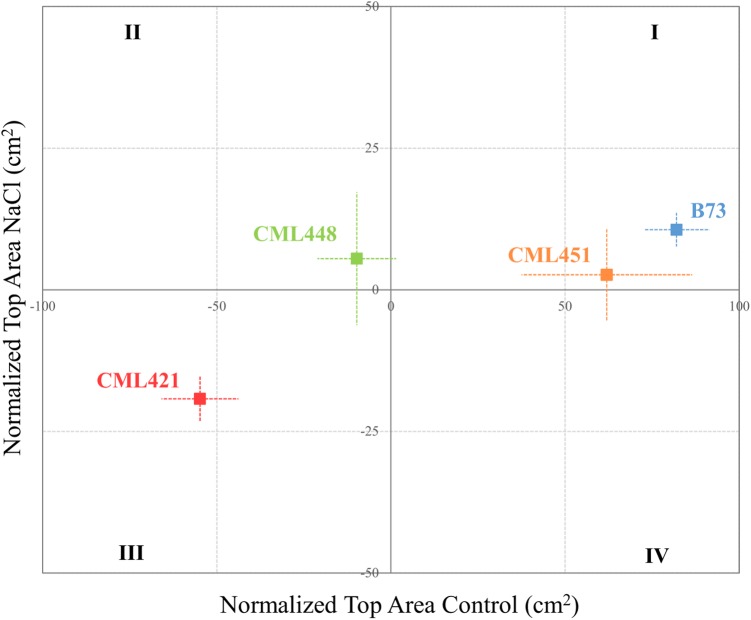
Quadrant rank of maize genotypes on the fourth week after sowing, according to the total median top area (cm^2^) for each soil condition. Vertical and horizontal lines discriminate the growth potential of the plants in soil with or without salt, respectively. Dotted lines represent standard error. *N* = 6.

**FIGURE 3:**
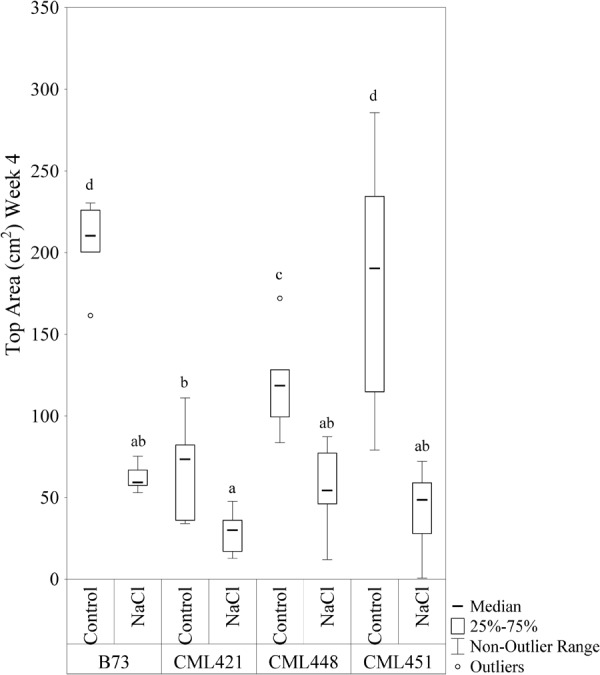
Top area (cm^2^) of four maize genotypes treated with NaCl or not (control), at the fourth week after sowing. Means followed by the same letter did not differ according to the LSD test (*p* < 0.05). *N* = 6.

**FIGURE 4:**
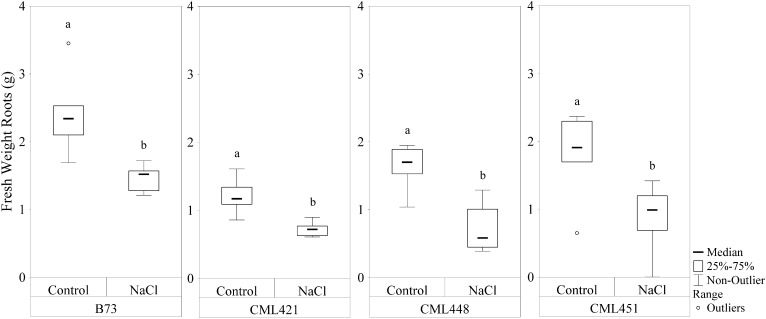
Fresh weight (g) of roots of four maize genotypes treated with NaCl or not (control), at the fourth week after sowing. Means followed by the same letter did not differ according to the LSD test (*p* < 0.05). *N* = 6.

Featured in the ranking quadrant (**Figure 2**), CML421 plants are situated in the third quadrant, which means that each individual of this genotype, at each treatment, had a lower leaf area than the median of all genotypes in the respective treatment. CML421 is characterized by the lowest growth in both conditions. B73 and CML 451 are represented in the first quadrant, which discriminates for relatively good growth and also displaying good vigor. The average leaf-area difference calculated between control and salt-stressed plants was biggest for genotype CML451 (76%), followed by B73 (70%), CML421 (57%) and CML448 (54%) (**Figure 3**). Root growth, determined by means of fresh weight at the fourth week (**Figure 4**) demonstrated a difference between treated and control plants, for all genotypes. Treated plants had significantly smaller roots than the ones not treated with salt and the average leaf-area differences are 39% for B73 and CML421, 46% for CML451 and 56% for CML448.

### Ion Content Determination

**FIGURE 5:**
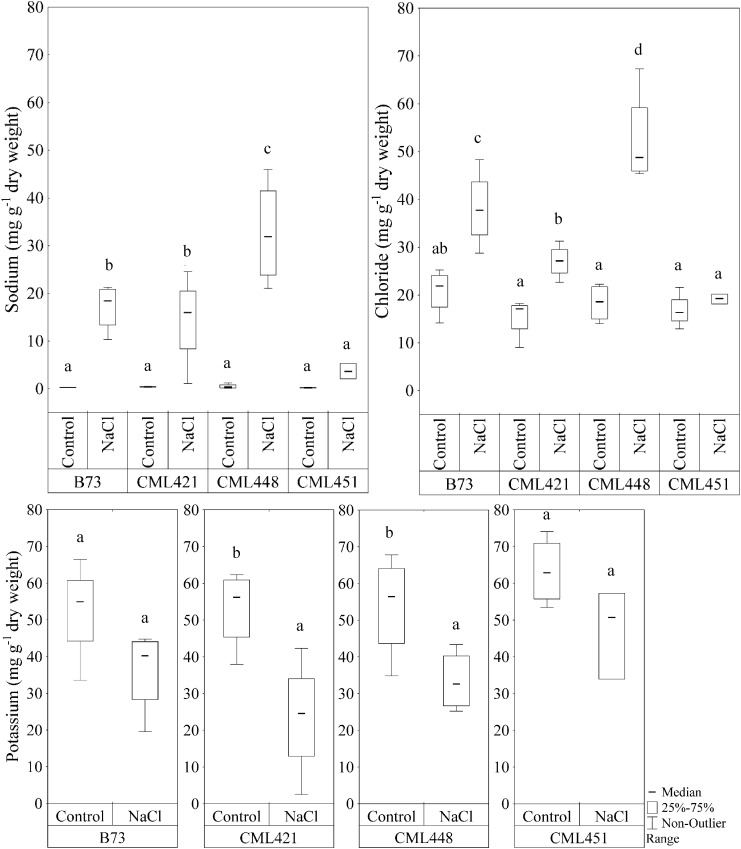
Sodium and chloride content (mg g^-1^ dry weight) in leaves of four maize genotypes, at the fourth week after sowing. Plants were grown in control or saline conditions (NaCl). Means followed by the same letter did not differ according to the LSD test (*p* ≤ 0.05), calculated separately for each ion. *N* = 4.

In the fourth week, sodium (Na^+^) and chloride (Cl^-^) showed a significant difference in the interaction of variables genotype and treatment (**Figure 5**). For the potassium concentration, there was a significant difference between control and treated plants for genotypes CML421 and CML448 (**Figure 5**).

There was a remarkable accumulation of both Na^+^ and Cl^-^ ions in the leaves of genotype CML448 grown in saline conditions, which was the most pronounced among all four genotypes. In average, sodium was 97% higher and chloride, 57%, in the salt treated plants of this genotype. In CML451, the Na^+^ and Cl^-^ concentrations were not significantly different between the NaCl-stress treatment and the control (**Figure 5**). Genotypes CML421 and CML448 exhibited a reduction in potassium content in salt treated leaves of 56 and 42%, respectively (**Figure 5**).

Salt stress negatively affects cell division and elongation of roots (Galvan-ampudia and Testerink, 2011), and monitoring their growth was proven to be an effective physiological marker for salt tolerance (De Azevedo Neto et al., 2004). For instance, the root epidermis plays a role in the perception of salt stress via mechanisms exerted by ABA in preventing root growth into soil sites with stressful levels of NaCl (Geng et al., 2013).

### Protein Identification and Responsiveness to Salt Stress

The reference time for proteomics sampling was determined by the physiological phenotyping. Thus, at the fourth week after sowing, the roots were collected and the protein content was investigated. In total, 1,747 proteins were identified (Supplementary Table S1.1), at a FDR of 0% calculated by Scaffold, minimum of one identified peptide and best ion score from 25 upward. From this, 403 were significantly influenced by the stress treatment: 209 proteins were more abundant in the stressed plants, while 194 were more abundant in roots of control plants (*p* < 0.05) (Supplementary Table S1.2).

A SEA was performed for these 209 and 194 differentially abundant proteins. Enriched proteins with high abundance to salinity had 29 significant shared GO terms, and the category Biological Process (BP), with 19 significant GO terms, is registered in the Supplementary Table S2. Enriched proteins with low abundance toward salt stress had 51 significant GO terms, from which 38 are in BP category (Supplementary Table S2).

For proteins less abundant in salt-stressed plants, the SEA showed that the major activated BPs categories were: (1) ‘cellular process,’ in which proteins are mainly involved in nucleosome assembly, (2) ‘catabolic process’ and (3) ‘cellular metabolic process,’ such as amino acid biosynthetic processes (Supplementary Table S2). As for salinity-activated BPs, the main categorization includes: (1) ‘catabolic process,’ (2) ‘metabolic process,’ where oxidation reduction processes and cellular respiration stood out, (3) ‘response to stress,’ including ‘response to oxidative stress’ and ‘response to water’ and (4) ‘biological regulation’ as ‘cell redox homeostasis’ (Supplementary Table S2).

**FIGURE 6:**
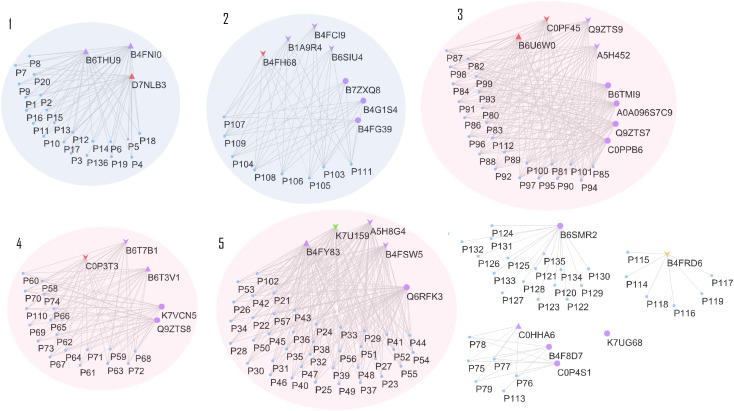
Cytoscape network relating class III peroxidase proteins and paralog genes. Proteins represented by purple triangles were positively correlated to salt stress, in all maize genotypes. Proteins represented by purple down arrows were negatively correlated to salt stress, for all maize genotypes Triangles and arrows in other colors than purple represents the class III peroxidases specifically differentiated to one genotype, being: colored red represents genotype CML421, green, genotype CML448 and orange, genotype CML451. Purple circles represent proteins that showed no difference in the relative abundance between control and treated plants. Clusters 1 and 2 gather proteins with no divergence in correlation to stress, while 3, 4, and 5, are groups that contain proteins negatively and positively correlated to stress. The paralog genes are displayed as a “P” plus a reference number, and their complete accession number can be found in Supplementary Table S3.

Effects on class III plant peroxidase (POX) abundances are noteworthy. In total, 34 peroxidases belonging to this class were confidently identified in the roots (Supplementary Table S3), from which seven were highly accumulated in stressed plants (B4FNI0, B4FY83, B6T3V1, B6THU9, C0HHA6, D7LNB3, and B6U6W0) and ten in control (A5H452, A5H8G4, B4FCI9, B6T7B1, K7U159, B1A9R4, B4FRD6, B65IU4, B4FSW5, and Q9ZTS9) (Supplementary Table S3). A compilation of all 34 class III peroxidases with their respective paralog genes, as well as their responsible gene location on the maize genome, are in the Supplementary Table S3. The class III peroxidase genes found in this work are spread throughout the 10 chromosomes (chr), except chr4 and chr9. In total, 136 paralog genes were related to the class III peroxidases and a network of paralogs and proteins was created using Cytoscape (**Figure 6**). This network aggregated class III peroxidases according to their shared connections of paralogs, forming eight groups and one lone protein (K7UG68). From these, six groups gathered two or more proteins, and within three of them, class III peroxidases presented divergence in correlation to salt stress, groups 3, 4, and 5 (**Figure 6**). To better understand this big family and to be able to interpret the abundance divergence of proteins even within the same group (**Figure 6**), promoter analysis was conducted, comparing the sequences of those clusters. We focused on stress-related promoter elements present in genes of the peroxidases positively induced by salt stress. For group 4, the most significant motifs are MBS, which constitutes an MYB binding site involved in drought-inducibility, and TC-rich repeats, a *cis*-acting element involved in defense and stress responsiveness (**Table 1**). In group 5, are present promoters ABRE (ABA responsive elements), which is a *cis*-acting element involved in the ABA responsiveness, DRECRTCOREAT (dehydration-responsive element/C-repeat), the core motif of DRE/CRT, and DRE2COREZMRAB17, a “DRE2” core found in maize rab17 gene promoter. Interestingly, in group 5 the promoter DRE2COREZMRAB17 is present exclusively in genes of salt stress positively induced class III peroxidase (B4FY83) (**Table 1**).

**Table 1:** Promoter region analysis of genes expressing class III peroxidases that belong to the same paralogs cluster (**Figure 6**) and presented different relative abundance toward salt stress.

Protein	Salt stress induction	Promoter position and strand
**Group 4 Class III peroxidasess**		
**Promoter: MBS**		
**Function: MYB binding site involved in drought-inducibility**		
B6T3V1	Positive	96 (+)
		1408 (–)
Q9ZTS8	Neutral	137 (+)
		1143 (+)
		787 (–)
		1030 (–)
K7VCN5	Neutral	1198 (–)
**Promoter: TC-rich repeats**		
**Function: *cis*-acting element involved in defense and stressresponsiveness**		
B6T3V1	Positive	1237 (–)
B6T7B1	Negative	42 (+)
Q9ZTS8	Neutral	718 (+)
**Group 5 Class III peroxidases**		
**Promoter: ABRE**		
**Function: *cis*-acting element involved in the abscisic acidresponsiveness**		
B4FY83	Positive	587 (+)
A5H8G4	Negative	940 (–)
		1042 (–)
		1378 (–)
**Promoter: DRECRTCOREAT**		
**Function: Core motif of DRE/CRT (dehydration-responsive element/C-repeat)**		
B4FY83	Positive	264 (–)
A5H8G4	Negative	917 (+)
**Promoter: DRE2COREZMRAB17**		
**Function: “DRE2” core found in maize (Z.M.) rab17 gene promoter**		
B4FY83	Positive	690 (–)

*Selected genes of proteins in groups four and five had their sequences analyzed using plantCARE online tool, up to 1,500 bp*.

### Proteins Differently Abundant in Response to Salt Stress, According to Genotype

Regarding the variability among the genotypes, 84 proteins were found to be significantly different: 52 in CML421 plants; 13 to CML451; 6 to CML448; and 5 to B73 (**Table 2**). Uncharacterized proteins were searched via BlastP tool option from Gramene^2^, in which the sequences data were searched against *Zea mays* protein database.

**Table 2:** Significant proteins differentially abundant to each maize genotype, in response to salt stress.

**Protein**	**Annotation**	**ANOVA**	**Number of identified peptides**	**Best ion score**	**Abundance in salt stress**
**List of significant proteins differentially abundant to genotype B73 (*p* < 0.05)**					
B4FTS6	Endochitinase A	1.5E-03	10	71.23	High
A0A096RHR4	Glutathione *S*-transferase 12	7.6E-03	8	57.86	Low
A0A096RY69	Glutathione transferase 11	8.2E-03	9	61.44	Low
A0A096SXV5	Probable alpha-mannosidase	2.0E-06	25	81.34	Low
K7U0Q4	Germin-like protein subfamily 1 member 8	3.2E-03	3	44.78	Low
**List of significant proteins differentially abundant to genotype CML421 (*p* < 0.05)**					
A0A096PV29	Protein EXORDIUM	7.9E-03	2	91.58	High
A0A096QCP1	Alpha/beta-Hydrolases superfamily protein	1.0E-02	5	67.3	High
A0A096QF11	TolB protein-related	1.3E-02	17	87.95	High
A0A096QHE8	Glutathione *S*-transferase L2 chloroplastic	1.7E-02	4	29.94	High
A0A096R6Z8	Heat shock 70 kDa protein 6 chloroplastic	4.0E-02	1	61.39	High
A0A096RB11	17.4 kDa class III heat shock protein	1.0E-03	1	37.33	High
A0A096RUZ6	Bowman-Birk type trypsin inhibitor	1.2E-02	9	38.25	High
A0A096S3X0	Succinate-semialdehyde dehydrogenase mitochondrial	1.8E-03	3	80.68	High
A0A096SQA7	Sarcosine oxidase	0.0E+00	8	73.2	High
A0A096SVP6	Octicosapeptide/Phox/Bem1p family protein	1.7E-02	3	45.02	High
A0A096TUS3	DPP6 N-terminal domain-like protein	2.4E-02	11	68.71	High
A0A096U7Y9	Adenosylmethionine aminotransferase 1	1.9E-03	6	102.82	High
A0A0A1P1P0	Cystatin protein	1.5E-02	2	32.5	High
A3KLI1	RAB17 protein	5.0E-04	9	66.41	High
B4F817	Dehydroascorbate reductase	1.6E-02	6	57.49	High
B4FI76	Adenylate kinase 4	1.3E-02	6	83.8	High
B4FPG2	Actin1 isoform 1	2.6E-03	6	74.27	High
B4FRS8	Germin-like protein subfamily T member 1	2.6E-03	4	55.02	High
B4G0K5	Hydroxyproline-rich glycoprotein family protein	0.0E+00	39	109.83	High
B5U8J9	Asparagine synthetase	7.0E-05	6	62.08	High
B6SIR9	1-Aminocyclopropane-1-carboxylate oxidase	3.0E-02	3	64.43	High
B6SLU8	Putative uncharacterized protein	1.6E-04	6	69.92	High
B6SZY7	Glutathione *S*-transferase IV	1.0E-02	7	68.29	High
B6T033	Glutathione *S*-transferase GSTU6	2.1E-03	6	78.68	High
B6T5H6	Auxin-induced protein PCNT115	2.2E-04	5	55.4	High
B6T916	*N*-acetyltransferase	1.5E-04	2	40.56	High
B6U9S6	APx1-cytosolic ascorbate peroxidase	4.1E-03	10	72.86	High
C0PJR9	Putative alcohol dehydrogenase superfamily protein	1.4E-04	2	55.27	High
C0PLV4	Dihydroflavonol-4-reductase	1.1E-02	1	54.66	High
C0PLX5	17.4 kDa class I heat shock protein 3	1.0E-05	1	68.53	High
K7U935	Catalase	9.2E-04	12	93.3	High
K7VB29	Tryptophan synthase beta type 2	5.0E-05	3	27.57	High
K7VES9	Asparagine synthetase	4.0E-02	13	71.36	High
K7VHA0	5-Pentadecatrienyl resorcinol *O*-methyltransferase	2.7E-02	1	34.54	High
A0A096PJN3	Heteroglycan glucosidase 1	1.5E-03	3	40.4	Low
A0A096T8I0	Cell number regulator 10	1.4E-02	1	52.01	Low
B4FH68	Peroxidase	3.5E-03	14	90.79	Low
B4FQE6	Uncharacterized protein	7.1E-03	1	45.13	Low
B4FVE1	NADPH quinone oxidoreductase 1	5.1E-03	13	96.74	Low
B6SWV1	Glucose-6-phosphate 1-dehydrogenase	8.0E-04	14	73.31	Low
B6T1G5	Histone H2B	3.0E-02	8	89.45	Low
B6T8I3	Disease resistance response protein 206	1.1E-04	3	87.74	Low
B6UHQ8	Blue copper protein	3.1E-03	2	51.37	Low
C0P3T3	Peroxidase	8.0E-05	5	106.75	Low
C0PF45	Peroxidase	2.5E-03	7	89.2	Low
C0PGU8	Glycerophosphodiester phosphodiesterase GDPDL3	8.2E-03	8	112.35	Low
C4J4U3	GDSL esterase/lipase	3.6E-03	8	87.71	Low
K7V6Z1	Putative patellin family protein	2.3E-04	2	44.36	Low
K7VEJ7	Nicotianamine synthase 1	2.4E-02	2	36.61	Low
M1H779	Lipoxygenase (fragment)	7.6E-03	1	44.4	Low
P51059	Phosphoenolpyruvate carboxylase 2	7.3E-03	3	22.92	Low
Q7XAT3	Glufosinate-resistant glutamine synthetase (fragment)	3.5E-03	1	71.18	Low
**List of significant proteins differentially abundant to genotype CML448 (*p* < 0.05)**					
A0A096QYG0	NAD(P)-binding Rossmann-fold superfamily protein	2.0E-09	21	103.76	High
A0A096Q155	Beta-glucosidase aggregating factor-like protein	3.1E-04	1	67.1	High
A0A096RTH6	Heat shock protein 90-2	4.6E-02	21	106.84	High
C0HF51	Zinc finger (C3HC4-type RING finger) family protein	2.9E-05	18	66.66	High
B4FS90	Cysteine protease 1	1.3E-03	10	98.91	Low
K7U159	Peroxidase	7.3E-03	5	32.28	Low
**List of significant proteins differentially abundant to genotype CML451 (*p* < 0.05)**					
B6SMC1	Thioredoxin	3.3E-03	3	49.05	High
B6T267	Ribosomal protein L15	9.1E-03	6	87.54	High
B6T2K5	60S ribosomal protein L35	1.4E-03	1	21.96	High
B6UH77	Histone H3	7.7E-03	11	56.75	High
C4J2M5	Pyruvate kinase	3.8E-03	5	101.31	High
A0A096QFM0	Guaiacol peroxidase 2	2.1E-03	6	71.84	Low
A0A096S9W1	Remorin	8.2E-04	5	30.12	Low
B4F935	Cystathionine gamma-synthase 1 chloroplastic	7.4E-03	5	43.29	Low
B4F9A4	NAD(P)-linked oxidoreductase superfamily protein	1.1E-03	16	63.73	Low
B6UGK8	3-isopropylmalate dehydratase small subunit 2	3.2E-02	4	67.87	Low
K7VC35	*S*-adenosylmethionine synthase	2.8E-03	18	66.87	Low
Q53WW9	Beta-D-glucosidase	8.2E-03	52	134.22	Low

**FIGURE 7:**
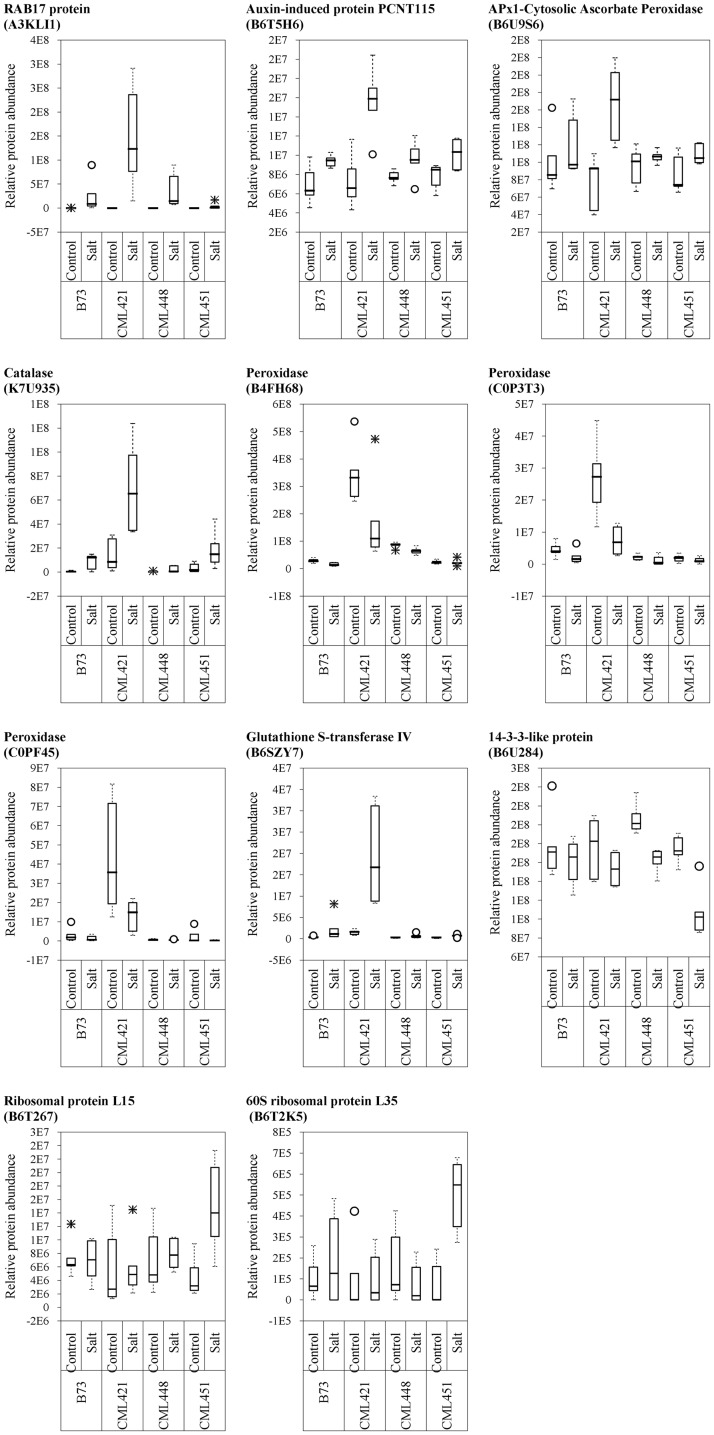
Variability plots of proteins selected by their genotype specificity and abundance toward salt stress. Relative abundance was calculated for the four maize genotypes, treated or not (control) with NaCl, at the fourth week after sowing.

From the 52 proteins differently accumulated in the slow growing genotype CML421, 34 were found to be highly abundant in response to salt stress and 18 lower abundant (**Table 2**). Salt stress abundant proteins for genotype CML421 are indicators of the stress impact, such as reactive oxygen species (ROS), heat shock proteins (A0A096R6Z8, A0A096RB11, and C0PLX5) and one dehydrin RAB17 protein (A3KLI1) (**Table 2** and **Figure 7**).

Genotype CML448 presented a heat-shock protein (HSP) 90-2 (A0A096RTH6), as possible evidence for the stress in salt-exposed plants (**Table 2**). One class III peroxidase (K7U159) was especially abundant in control plants of this genotype (**Table 2**).

Genotype CML451 presented a higher abundance of proteins involved in gene and protein regulation in treated plants (**Table 2**). Obviously, they are also found in other genotypes, but the relative abundance was the highest for CML451 in saline soil. Other possible salt-related response proteins had higher abundance in the control compared with salt-treated plants for this genotype, the 14-3-3-like protein (**Figure 7**).

Five class III peroxidases reacted specifically to CML421 toward salt stress: C0P3T3, B4FH68, and C0PF45 were lower in salt-stressed plants, and B6U6W0 and D7NLB3 were more abundant (**Figure 7**). Other specific peroxidases that reacted negatively to salt stress were K7U159 in plants of genotype CML448 and B4FRD6, in CML451.

## Discussion

### Growth Impact Caused by Salt Stress

The roots are the plant organs that first encounter salt in a saline environment. From there, signals that communicate the onset of salt stress spread systemically toward cells and organs that may be able to respond and anticipate. However, when the roots are hidden under the soil, the outcomes caused by salt stress are revealed to us in the aerial portion of the plant. In this work, the monitoring of the physiological changes through images proportioned us a dynamic phenotyping, which led to the ranking of four maize genotypes regarding differing salt stress tolerance (**Figure 2**).

It is broadly known that salinity slows down leaf growth rates, either by transient osmotic imbalance on the cells, reduction of the cell elongation and division (Fricke and Peters, 2002) and/or cell wall stiffening (Geilfus et al., 2017). The slower growth of shoots was confirmed in our results (**Figure 3** and Supplementary Figure S2). Although the stressed plants accumulated high amounts of ions in their leaves, they were still able to grow (**Figure 3**).

The growth limitation of plants in osmotic and ionic stress can be caused by an energy shortage due to a photosynthesis reduction or energy relocation into defense mechanisms (Munns and Gilliham, 2015). The growth maintenance, even if decelerated, requires sources of energy and thus, an increase in respiration can be induced in such circumstances (Zorrilla-fontanes et al., 2016). In our work, the salt treatment affected all genotypes and their root growth was significantly slower in comparison to control plants (**Figure 4**). Concomitantly, the same stressed plants presented a higher activation of processes involved in homeostasis regulation, response to oxidative stress, response to water stress and cellular respiration. The pathways involved in cell division and biosynthetic processes were slowed down (Supplementary Table S2). Therefore, we reinforce the hypothesis of an increased energy demand related to stress and the subsequent enhanced respiration rates.

### LEA Proteins as Markers of Salt Stress

We identified five different proteins belonging to the *Late embryogenesis abundant* proteins (LEA proteins) (B4F9K0, A3KLI0, A3KLI1, B7U627, and C4J477), in response to salinity (Supplementary Table S1.2). Those play a role in acclimatization to stresses related to dehydration (Hong-Bo et al., 2005). LEA proteins were found to be produced during dehydration and they are predicted to protect structures and molecules. In this way, they can contribute to coping with drought and salt stress in different plant species (Chen et al., 2002; Saavedra et al., 2006; Brini et al., 2007). Considering their function, they were suggested as excellent molecular markers for water stress (Hanin et al., 2011). Whether they are effective markers for drought tolerance is another issue.

### Antioxidative Responses

Gene ontology enrichment ‘response to oxidative stress’ and ‘cell redox homeostasis’ for proteins highly abundant in salt stress gathered several ROS scavenger proteins: class III peroxidases (B6T3V1, B4FNI0, B6THU9, C0HHA6, B4FY83), APx1-cytosolic ascorbate peroxidases (B6U9S6, B6UB73), glutathione peroxidase (Q6JAH6), APx4-peroxisomal ascorbate peroxidase (B4FA06), protein disulfide isomerase (Q5EUE1, Q5EUD6, A5A5E7), Grx_C2.2-glutaredoxin subgroup I (B4FXZ3), thioredoxin h1 protein (Q4W1F7), and peroxiredoxin-5 (B6THT0). Those antioxidants are efficient fighters of ROS found in response to different environmental stresses, including salinity (Sharma et al., 2012). Sairam et al. (2002), working with wheat, demonstrated that their salt tolerant genotype had a lower decrease in biomass and grain yield, allied with a higher antioxidant activity when grown in saline conditions. Thus, the activity of such detoxifying proteins is one of the mechanisms encountered in salt stressed plants contributing to their acclimation in that environment.

### Class III Plants Peroxidase

Class III peroxidases are enzymes secreted into the apoplast, where they oxidize (take electrons from donor molecules) phenolics, lignin precursors or other secondary metabolites. They also oxidize the growth hormone auxin, as well as other substrates (Gaspar et al., 1982) and produce hydrogen peroxide (Blee et al., 2001) and hydroxyl radicals (Chen and Schopfer, 1999), two activated oxygen species involved in oxidative burst and in cell elongation. It is a multigenic complex family (Hiraga, 2001), with roles in several plant developmental processes, including regulation of cell elongation, cell wall construction and defense against pathogens (Mika et al., 2004; Passardi et al., 2004a). Cell wall stiffening is controlled by the cross-linkage of the peroxidase with compounds of the wall, such as extensin, lignin subunits and phenol molecules of the suberin constitution (Passardi et al., 2004b). Class III peroxidases can be induced by environmental stresses, participating direct- or indirectly of the plants responses (Cosio and Dunand, 2009).

Maize has 158 class III peroxidases (Koua et al., 2009). The complexity of diverse physiological roles and the number of genes suggests that each enzyme isoform went through a functional specialization (Cosio and Dunand, 2009). In this work, we present 34 class III peroxidases expressed in the roots, with peroxidases positively (B4FNI0, B4FY83, B6T3V1 B6THU9, C0HHA6, D7LNB3, and B6U6W0) and negatively (A5H452, A5H8G4, B4FCI9, B6T7B1, K7U159, B1A9R4, B4FRD6, B65IU4, B4FSW5, and Q9ZTS9) correlated to salt stress (Supplementary Table S1.2 and **Figure 6**). The function of those class III peroxidases negatively correlated to salt stress isoforms is predicted to be involved in cellular growth and elongation. The peroxidase isoforms positively correlated to stress, may be acting in the acclimation to salinity, e.g., helping in stiffening the root cell wall. This could be relevant to prevent root growth toward salinized soil sites.

The 34 class III peroxidases identified belong to a group of 136 paralog genes connections, forming eight groups (**Figure 6**). Despite it is expected that closely related paralog groups have similar expression patterns and functionality (Du et al., 2012), we detected divergent abundance patterns within three clusters (**Figure 6**). By analyzing the promoters of divergent groups, we found that the MBS and TC-rich repeats motifs are in different positions and strands for the proteins of group 4 (**Table 1**). This difference in the promoter region might be a factor leading to a differential expression of these genes. In group 5 (**Table 1**), the ABRE and DRECRTCOREAT motifs were predicted. They are located in different positions and strands of the promoter regions. Remarkably, the motif DRE2COREZMRAB17 is present only in the promoter region of a gene expressing positively a peroxidase (B4FY83) to salt stress. This C-repeat/DRE motif is involved in dehydration and salinity stress processes, and it is participative of ABA induction in maize (Kizis, 2002). The presence of this motif in the promoter region of the salt-induced peroxidase B4FY83 might explain its higher relative abundance.

### Genotype-Specific Reactions

We can say that despite that all genotypes started in similar conditions, they did not have the same salt-acclimatization level nor the same stress level, evidenced by the differential ion concentration in leaves (**Figure 5**), and the different leaf area and root FW produced under stress conditions (**Figures 2, 4**). Under stress, genotype CML448 showed a similar growth while it accumulated significantly more Na^+^ and Cl^-^ ions (**Figure 5**). This indicates that CML448 tolerates higher amounts in its leaves and might have mechanisms for the partitioning of those ions away from the cytosol or away from the primary site of the photosynthesis and growing tissues.

Proteins APx1 cytosolic ascorbate peroxidase (B6U9S6), catalase (K7U935), RAB17 protein (A3KLI1), and glutathione *S*-transferase IV (B6SZY7) were found as a response to salinity in all genotypes, but for CML421 they were present in a higher abundance (**Figure 7**). Besides that, three HSPs (A0A096R6Z8, A0A096RB11, and C0PLX5) were also found in higher abundance in salt-stressed plants of this genotype (**Table 1**). HSPs have the important role of protein folding, assembly and translocation in plants under optimal and stress conditions. Their specific major function during abiotic stress includes aggregation prevention and stabilization of non-native proteins, as well as the following of signaling processes leading to the synthesis of stress-response proteins (Wang et al., 2004). Vanhove et al. (2015) identified a specific osmotic responsive HSP70 isoform, evidencing that the behavior of paralogs are not always similar toward a stress condition. Thus, for genotype CML421, the strongest response to ROS and dehydration, is displayed by the slowest growth under stress, and might indicate a higher stress level (**Figure 2**).

14-3-3-like protein (B6U284) was remarkably lower abundant in salt-stressed plants of genotype CML451 (**Figure 7**). This protein family is known for its role in controlling K^+^ channels (Van Den Wijngaard et al., 2005). It is also a key activator of H^+^-ATPase activity (Fuglsang et al., 1999). We make a link between the lowest abundance of a 14-3-3-like protein (B6U284) in the roots of genotype CML451 and its remarkable low Na^+^ concentration and no alteration of potassium content in leaves of salt stressed plants (**Figure 5**). However, despite the fact that this genotype was able to avoid accumulating toxic ion contents in its leaves, this behavior has not translated into superior growth under stress (**Figure 3**).

Two ribosomal proteins were more abundant in response to salinity in plants of genotype CML451 (B6T267 and B6T2K5) (**Figure 7**). Abundance changes of proteins involved in protein synthesis have been reported in response to salt stress, including ribosomal proteins (Zörb et al., 2004; Pang et al., 2010; Omidbakhshfard et al., 2012). The role for ribosomal protein synthesis during periods of abiotic stress would be to maintain and regenerate plant protein synthesis mechanisms (Zörb et al., 2004). Under stress conditions, proteins assume important functions that confer enhanced tolerance to the plants, such as antioxidative activity, LEA proteins in osmotic adjustment, ion transporters, among several others (Kosová et al., 2013). Thus, the increased capacity to produce new proteins is directly related to the capacity to overcome salinity effects. Furthermore, Li et al. (2015) demonstrated that the regulation of vacuolar trafficking is likely to involve ribosomal proteins. As genotype CML451 presented the lowest leaf ion accumulation in salt treated plants (**Figure 5**), we speculate that they were compartmentalized in roots vacuoles, a strategic salt tolerance mechanism (Munns and Gilliham, 2015).

Three class III peroxidases (B4FH68, C0P3T3, and C0PF45) were significantly more abundant in genotype CML421 (**Figure 7**). They belong to three different subfamilies of the maize class III peroxidases (**Figure 6**). Cosio et al. (2008) demonstrated a negative regulation of a courgette class III peroxidase toward auxin, which led to growth suspension of the hypocotyl. Thus, the higher abundance of these three peroxidases in genotype CML421 may explain the slow general growth of this genotype (**Figures 4, 6**).

## Conclusion

This work has shown that phenotyping offers a relevant strategy to determine a good timing to conduct proteomics analyses that help elucidate stress-responses. The study also demonstrates that phenotyping is also very useful for characterizing geno- and phenotype-specific stress-responses. Salinity is a complex form of stress, and tolerance is a multigenic trait, with several origins and levels. Tolerance is also a complex concept that is difficult to define.

Despite this challenging context for such stress-response studies, the work has revealed significant genotype-specific stress responses in maize, and also some elements of the underlying stress-response mechanisms. In this study, genotype CML448 tolerated the highest ion content in its leaves whilst still able to grow, suggesting some level of stress tolerance. However, CML 448 also displayed reduced vigor under control conditions. Genotype CML451 avoided accumulation of ions in its shoots and displayed relatively strong vigor. Lower concentrations of a potentially channel-regulating protein and a higher abundance of proteins acting in protein synthesis and possible ion compartmentation were recorded in CML 451. This might explain the low-ion accumulation phenotype. Genotype CML 421 is the worst performing, since it displayed low vigor. The CML 421 genotype stress-specific proteins are related to the anti-oxidative system and responses associated with dehydration. These proteins indicate that this genotype was more stressed despite all genotypes being exposed to the same saline conditions. A rigid classification of the genotypes as salt-tolerant is not possible and needs to be considered for each agro-ecological target area. Therefore, we ranked all genotypes according to their growth under both control and stressed conditions so that the general vigor can be determined. Further analyses are needed to elucidate hidden links of salt tolerance and acclimatization mechanisms.

## Author Contributions

AS and SC designed the experiments. AS performed the experiments and compiled the data. SC supervised the experiments and conceived the project. AS, SC, and C-MG reviewed the results and wrote the article. All authors contributed to manuscript revision, read and approved the submitted version.

## Conflict of Interest Statement

The authors declare that the research was conducted in the absence of any commercial or financial relationships that could be construed as a potential conflict of interest.

## Footnotes

1 http://bioinfo.cau.edu.cn/agriGO/analysis.php

2 http://www.gramene.org/
