# Supplementary figures and images for: Genotype-Specific Growth and Proteomic Responses of Maize Toward Salt Stress
Source: Front Plant Sci. 2018 May 30;9:661. doi: 10.3389/fpls.2018.00661 (PMC5989331; doi:10.3389/fpls.2018.00661)

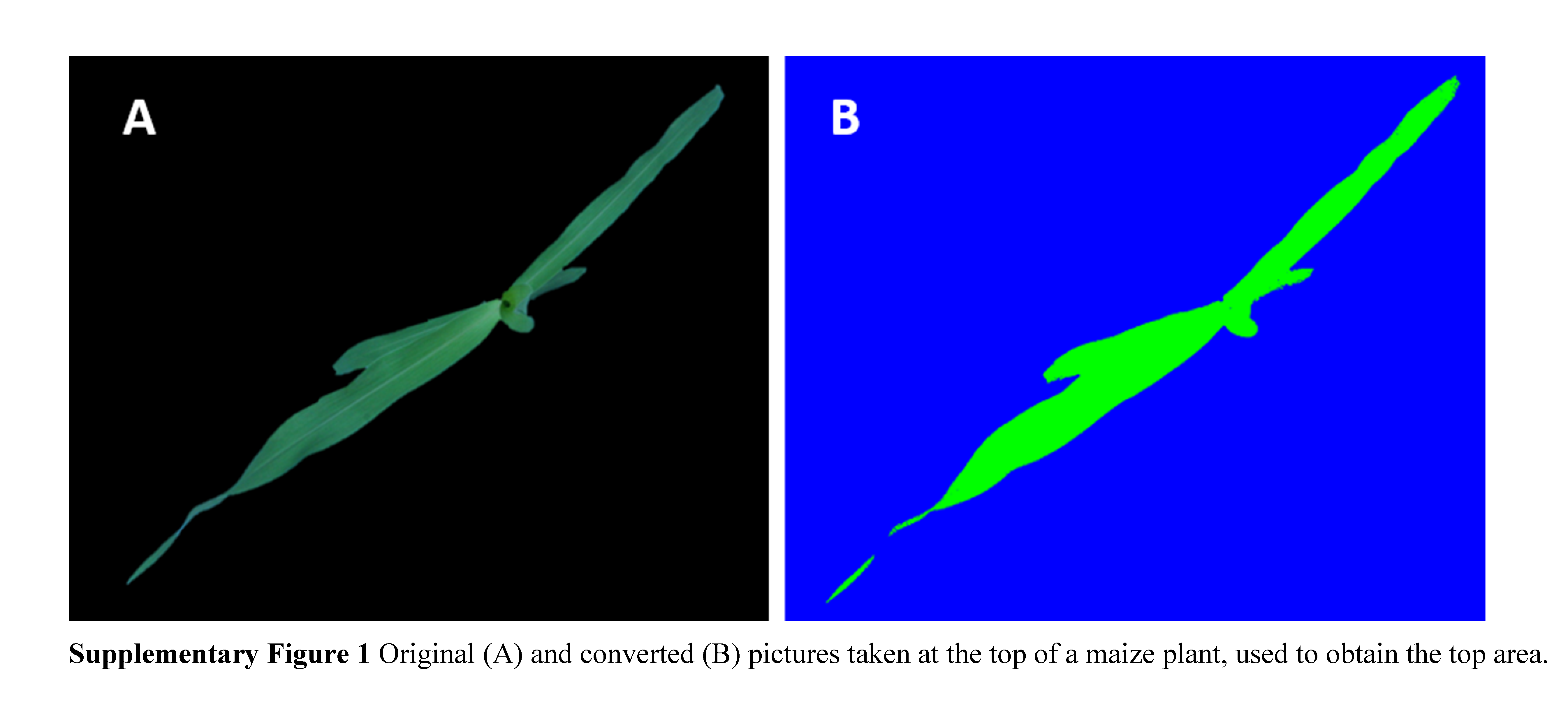

Supplement: Supplementary file 1 [file Image_1.TIF]

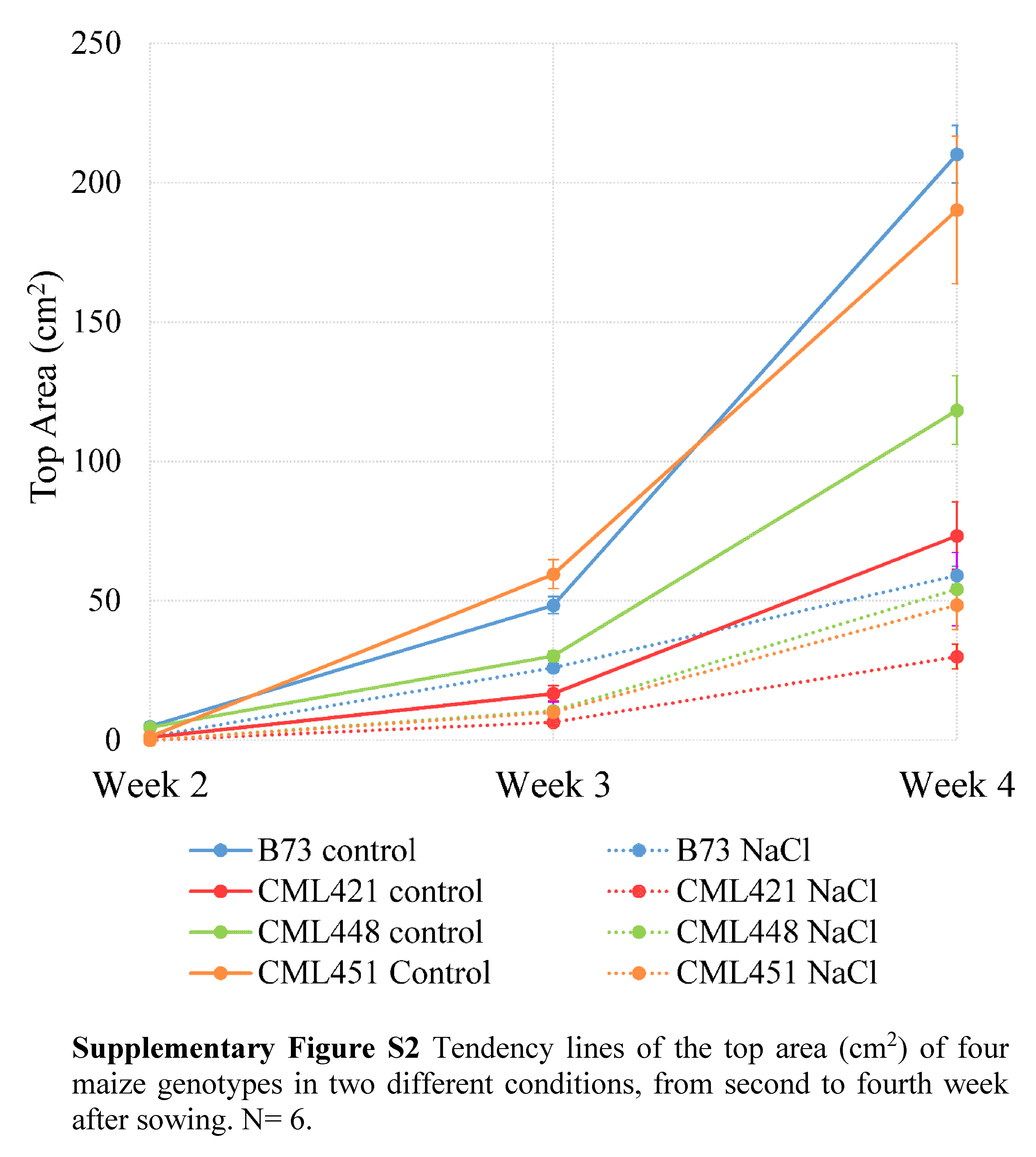

Supplement: Supplementary file 2 [file Image_2.TIF]
